# Senescence-related deterioration of intercellular junctions in the peritoneal mesothelium promotes the transmesothelial invasion of ovarian cancer cells

**DOI:** 10.1038/s41598-019-44123-4

**Published:** 2019-05-20

**Authors:** Martyna Pakuła, Anna Witucka, Paweł Uruski, Artur Radziemski, Rafał Moszyński, Dariusz Szpurek, Konstantin Maksin, Aldona Woźniak, Stefan Sajdak, Andrzej Tykarski, Justyna Mikuła-Pietrasik, Krzysztof Książek

**Affiliations:** 1 Department of Hypertensiology, Angiology and Internal Medicine, Poznań University of Medical Sciences, Długa 1/2 Str., 61-848 Poznań, Poland; 2 Division of Gynecological Surgery, Poznań University of Medical Sciences, Polna 33 Str., 60-535 Poznań, Poland; 3 Department of Clinical Pathology, Poznań University of Medical Sciences, Przybyszewskiego 49 Str., 60-355 Poznań, Poland; 4 Present Address: Department of Cosmetology, University of Information, Technology and Management in Rzeszow, Kielnarowa 386a Str., 36-020 Tyczyn, Poland

**Keywords:** Ovarian cancer, Metastasis

## Abstract

Mechanisms of transmesothelial invasion of ovarian cancer are still poorly understood. Here we examined whether this phenomenon may be determined by an expression of intercellular junctions in peritoneal mesothelial cells (PMCs). Analysis of ovarian tumors showed that cancer cells are localized below an intact layer of PMCs. The PMCs located near the invaded cancer cells displayed low expression of connexin 43, E-cadherin, occludin, and desmoglein, as well as expressed SA-β-Gal, a marker of senescence. Experiments *in vitro* showed that senescent PMCs exhibited decreased levels of the four tested intercellular junctions, and that the invasion of ovarian cancer cells through the PMCs increased proportionally to the admixture of senescent cells. Intervention studies showed that the expression of connexin 43, E-cadherin, occludin, and desmoglein in senescent PMCs could be restored upon the blockade of p38 MAPK, NF-κB, AKT, JNK, HGF, and TGF-β1. When these molecules were neutralized, the efficiency of the transmesothelial cancer cell invasion was diminished. Collectively, our findings show that the integrity of the peritoneal mesothelium, which is determined by the expression of junctional proteins, is critical for the invasion of ovarian cancer. They also indicate a mechanism by which senescent PMCs may promote the invasive potential of cancer cells.

## Introduction

Epithelial ovarian cancer (EOC) remains the most fatal malignancy of the female genital tract. The most devastating stage of the disease and, at the same time, a sign of poor prognosis is when the cancer progresses within the peritoneal cavity. Even after radical cytoreduction followed by treatment with platinum derivatives and/or taxanes, the disease tends to recur, often as a chemoresistant variant^1–3^.

The pathophysiology of the intraperitoneal spread of ovarian cancer is one of the most extensively studied aspects of the disease^4^. There is a consensus that the interaction between cancer cells that shed from the surface of the ovary, individually or in spheroids, and peritoneal mesothelial cells (PMCs) that cover, as a single layer, the visceral and abdominal surfaces of the peritoneum is the basis of tumor development. This cross-talk includes several individual processes, of which adhesion, migration, proliferation, and invasion are the most critical. They are accompanied by some supportive phenomena, such as the epithelial-mesenchymal transition of cancer cells and angiogenesis^5^. Additionally, a significant role is played by the malignant ascites, a fluid that accumulates within the peritoneum in a large number of patients with EOC^6^. The last decade also provided evidence that the cancer-promoting properties of PMCs are significantly reinforced when they become senescent. Senescent PMCs have been found to accumulate within the peritoneal cavity, and their supportive role in the development of an intraperitoneal metastatic niche has been observed in cell culture, laboratory animals, and tumors from EOC patients^7^.

Among the several steps of intraperitoneal ovarian cancer progression, the transmesothelial invasion of cancer cells towards the tissue stroma is the step whose molecular mechanism still needs to be clarified. In general, invasion refers to a process in which cancer cells actively move in response to chemotactic stimuli through the cellular and acellular (extracellular matrix) tissue compartments^8^. The study presented here stems from our observation of ovarian cancer cells lying beneath an intact layer of PMCs, suggesting that their invasion may be determined by expression of intercellular junctions in the PMCs. To verify this theory, we examined the expression of four junctional proteins that are constitutively expressed by PMCs: connexin 43 (gap junction), E-cadherin (adherens junction), occludin (tight junction), and desmoglein (desmosomes) in peritoneal ovarian tumors and in PMCs *in vitro*. Moreover, we showed that transmesothelial invasion of ovarian cancer cells is intensified by the presence and altered phenotype of senescent PMCs. The phenomenological aspects of the study were followed with mechanistic insights into the signaling pathways and molecules linked with cellular senescence that could be involved in the senescence-related deterioration of intercellular junctions, leading to intensified cancer cell invasiveness.

## Results and Discussion

There is a theory that the PMCs serve as a mechanical barrier that protects the peritoneal cavity from the entrance of ovarian cancer cells^9^. In this view, the PMCs are actively cleared by cancer cells, plausibly via myosin-driven forces, which allows the latter to successfully implant within the peritoneal stroma, where they are encouraged by fibroblasts to multiply and form tumors^10^. We are skeptical of this theory because our analysis of several samples of peritoneal ovarian tumors that developed in patients and laboratory animals revealed that the layer of PMCs was consistently intact (the cells were visualized using the D2-40 antigen in^11,12^ and the Wt-1 antigen in^13^).

In our opinion, the microphotographs provided by Kenny *et al*.^14^, and showing the lack of PMCs in proximity to ovarian cancer cells that have been used to support the theory mentioned above have been misinterpreted. In fact, the PMCs were still present below the implanted cells but seem to have moved slightly downward. More importantly, the pictures show cancer cells that had not invaded the PMCs into the peritoneal stroma and that are only attached to the external side of the omentum^14^.

Recently, the view that PMCs are “the first line of defense” against tumors metastasizing into the peritoneum and that they inhibit some vital processes underlying peritoneal dissemination (e.g., adhesion)^15^ has shifted towards an appreciation of the PMCs as cells that support ovarian cancer spread^16^. The microphotographs of cancer cells that were still above the PMCs, far from the tissue stroma^14^, directed, however, our attention to the issue of the mechanism controlling the transmesothelial invasion of the cancer cells.

**Figure 1:**
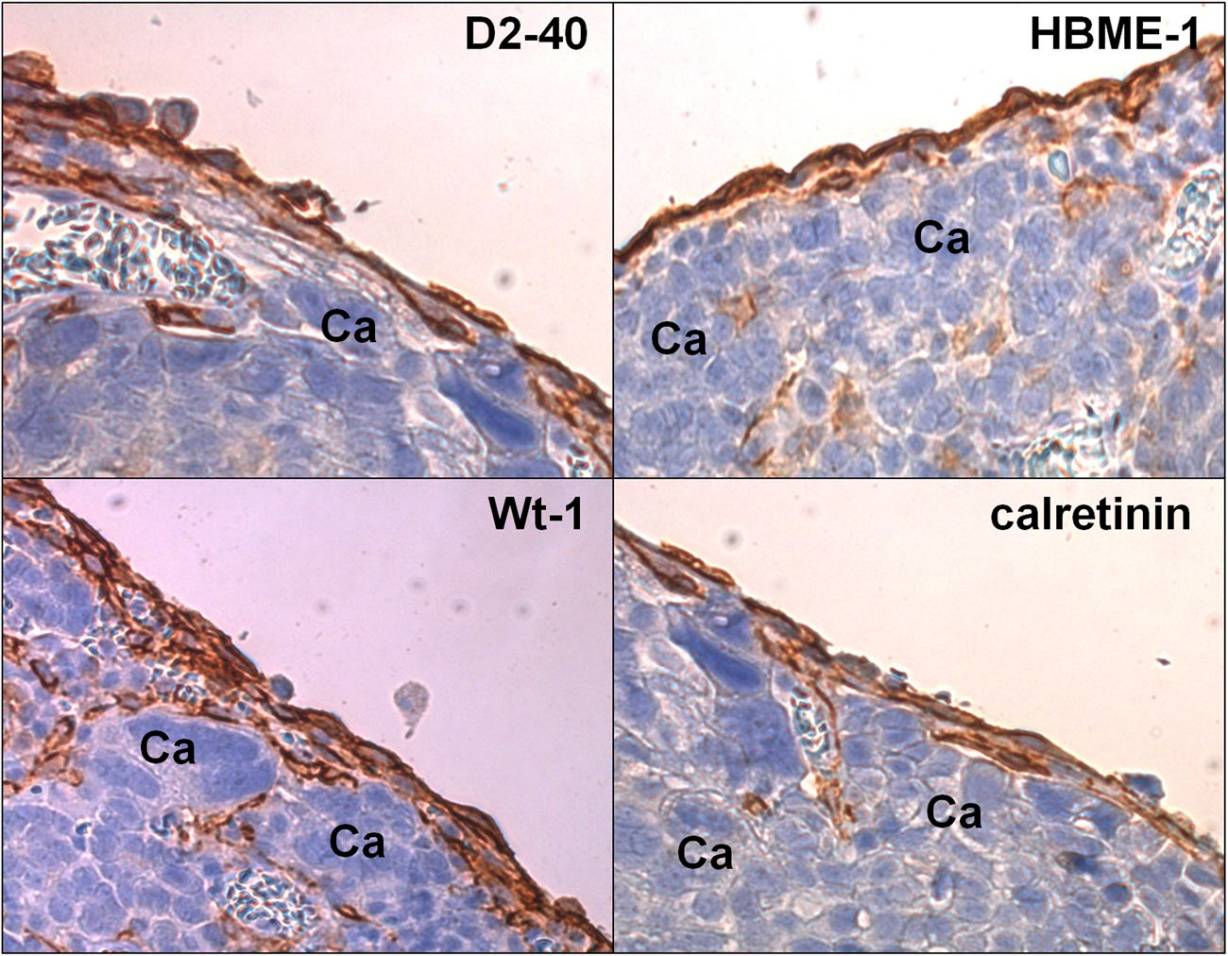
Representative pictures showing the presence of a uniform layer of PMCs in the biopsies in which ovarian cancer cells invaded the tissue stroma. The PMCs were identified according to brown staining of D2-40, Wt-1, HBME-1, and calretinin (magnification ×100). Ca – cancerous tissue. Analysis was performed using 32 tumors from patients with EOC.

Our analysis of 32 peritoneal ovarian tumors from the perspective of the histological organization of the cancer cells showed that cancer cells are consistently localized below an intact layer of the PMCs. The integrity of the PMCs that were effectively invaded by cancer cells was confirmed by using four different markers of these cells, that is D2-40, Wt-1, HBME-1, and calretinin (Fig. 1).

The observation that cancer cells penetrated the PMCs without their retraction raised the question about the plausible mechanism of this process. In this context, our attention attracted an interesting proposal that suggested the role of intercellular junctions as the gates by which ovarian cancer cells can pass through the PMCs. The authors of that study used scanning electron microscopy and immunohistochemistry to show that ovarian cancer cells may disrupt the intercellular junctions within the PMCs^17^. Over the next few years, a plethora of data has accumulated that various types of intercellular junctions are important for the invasion of cancer cells^18^. To verify whether this relatively old observation could explain our current findings, we examined the expression of four arbitrarily selected junctional proteins that are constitutively expressed by PMCs, connexin 43^19^, E-cadherin^20^, occludin^21^, and desmoglein^22^. The proteins were analyzed in either samples of tumors, tumor-free zones of the peritoneum, or cultured PMCs.

**Figure 2:**
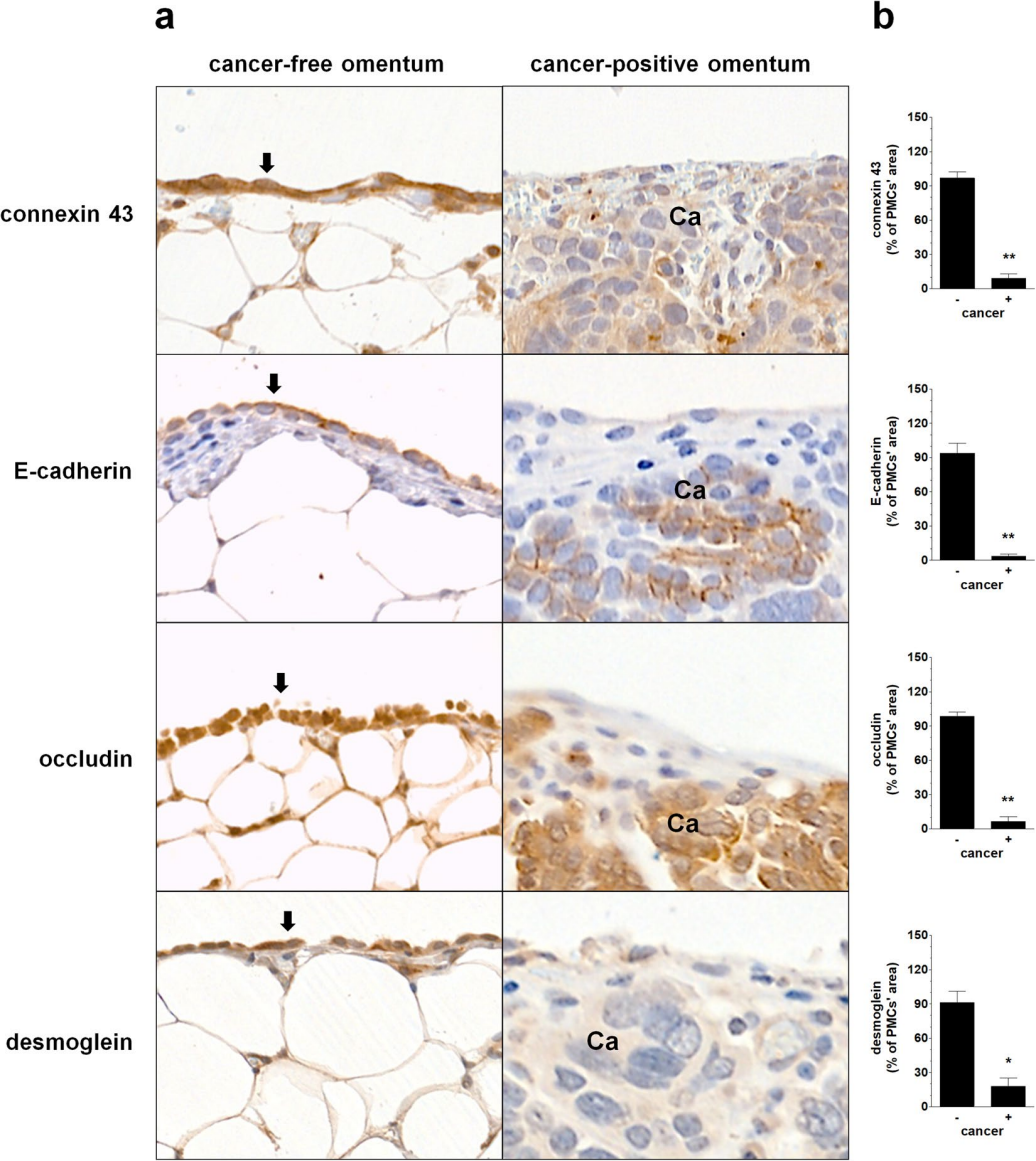
Representative pictures showing the expression of the intercellular junctions in the PMCs located near ovarian cancer cells that had invaded them and had reached the stroma and in the areas of the peritoneum free from cancer cells. (**a**) The arrows indicate a layer of PMCs (magnification ×100). Ca – cancerous tissue. Panel (b) shows a quantification of the junctional protein expression in the examined tissues. Analysis was performed using 32 tumors from patients with EOC. The results are expressed as the mean ± SEM. *P < 0.05; **P < 0.01 vs. cancer (−).

The analysis of the junctional proteins in tumor specimens revealed that in the case of cancer cells that penetrated the PMCs and settled in the tissue stroma, the expression of the junctional proteins in the PMCs was almost undetectable. In contrast, fragments of the omentum that were free of cancer cells displayed very high expression of the tested junctional proteins (Fig. 2). This result may suggest that cancer cells may freely pass through the PMCs when the expression of the intercellular junctions in the PMCs markedly declines, which plausibly corresponds to their decreased structural integrity (recognized by some as their retraction^10^) and increased penetrability. At the same time, we can speculate that when the functionality of the junctions is preserved, cancer cells are, in turn, unable to go to the stromal compartment. Probably, they can still form tumors; however, those lesions are deprived of the stimuli generated by stromal cells, particularly fibroblasts^23^.

**Figure 3:**
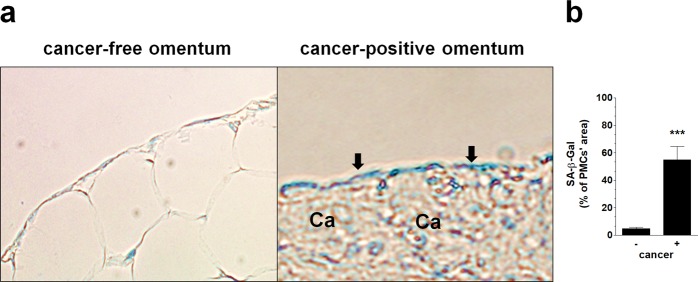
The presence of senescent PMCs in proximity to the cancerous tissue. (**a**) The senescent cells were identified according to green staining of SA-β-Gal. Panel (b) shows a quantification of the SA-β-Gal-positive cells in the examined tissues. Analysis was performed using 32 tumors from patients with EOC. The results are expressed as the mean ± SEM. ***P < 0.001 vs. cancer (−).

It has recently been shown that the expression of connexin 43 in PMCs located near cancer cells correlated with the presence of senescent PMCs^12^. There is also evidence that senescent PMCs accumulate in the peritoneum *in vivo*^24^. In the current study we extended those findings by showing that senescent, SA-β-Gal-positive cells are present in all of 32 tested peritoneal ovarian tumors, and that the fraction of these cells in the proximity to cancerous tissue ranges from 44 to 67%, and is significantly higher compared with tumor-free zones of the omentum (Fig. 3). This abundance of senescent PMCs within ovarian tumors strongly supports our previous *in vitro* and *in vivo* experiments, in which we demonstrated an explicit cancer-promoting activity of these cells. In fact, we were able to demonstrate that senescent PMCs support all vital elements of ovarian cancer progression, including adhesion, proliferation, migration, and angiogenesis^11,25,26^. Moreover, taking into account that the fraction of senescent PMCs in the omentum increases progressively during aging^24^, it is very plausible that these cells may significantly contribute to the age-dependent increase in ovarian cancer cell aggressiveness^27^.

**Figure 4:**
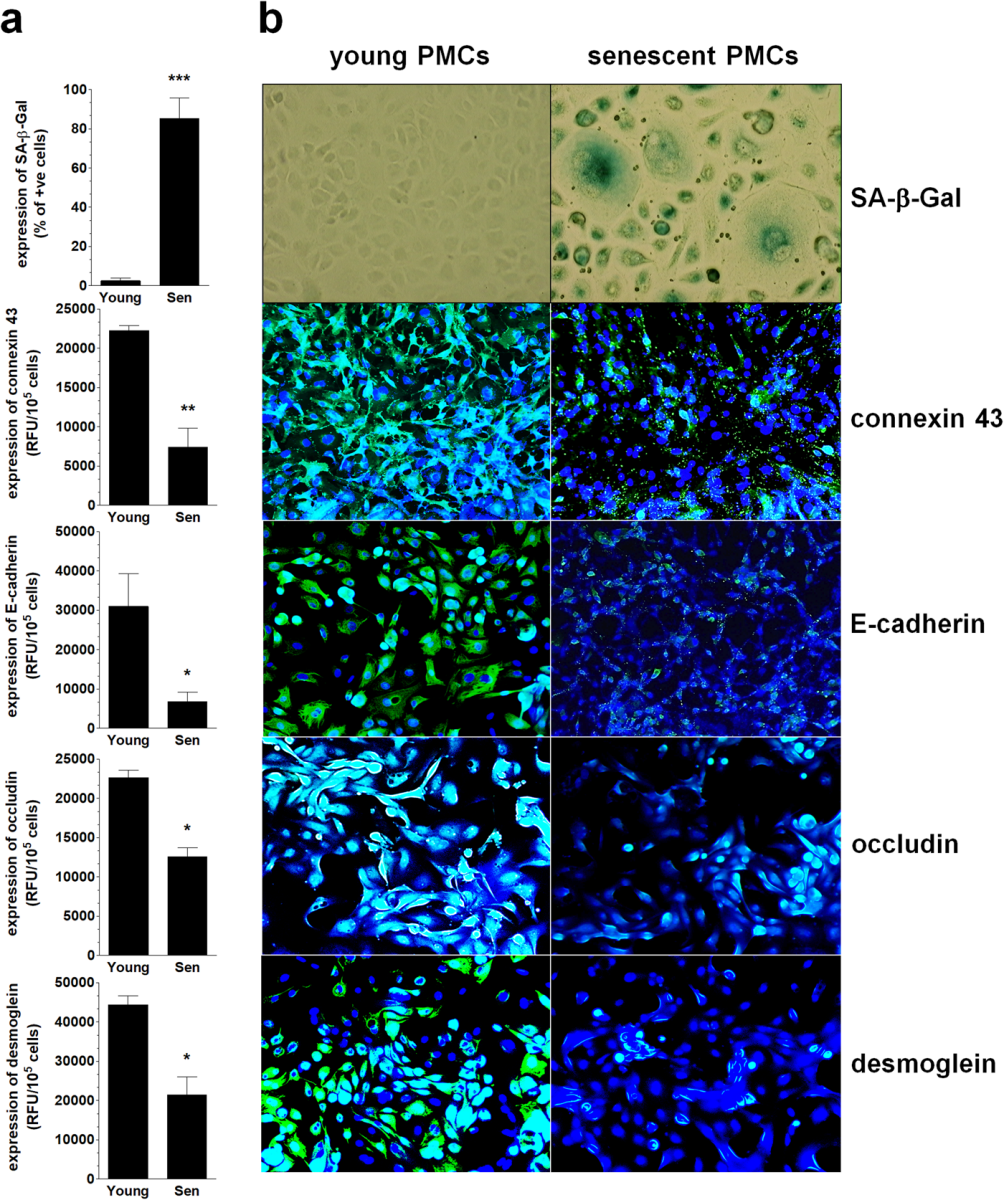
Changes in the expression of intercellular junctions during the senescence of PMCs *in vitro*. The results of the quantitative examination of SA-β-Gal, connexin 43, E-cadherin, occludin, and desmoglein in young (Young) and senescent (Sen) PMCs. (**a**) Representative pictures showing the decreased expression of junctional proteins in the senescent PMCs. (**b**) Experiments were performed with 8 cultures of PMCs obtained from different donors. The results are expressed as the mean ± SEM. *P < 0.05; **P < 0.01; ***P < 0.001 vs. Young. RFU – Relative Fluorescence Units (magnification ×200).

The above-mentioned observations, connecting the decreased expression of connexin 43 with the presence of senescent PMCs and the submesothelial localization of ovarian cancer cells, prompted us to examine the effect of PMC senescence on the invasion of cancer cells. To this end, we analyzed whether replicative senescence of PMCs is associated with changes in expression of connexin 43 and three remaining junctional proteins. Results of our investigations, depicted on Fig. 4 revealed that the expression of the four tested intercellular junctions in the senescent PMCs is considerably lower than in their younger counterparts. Recently, a senescence-associated decline in connexin 43 levels has been found in senescent fibroblasts^28^ and endothelial cells^29^. Senescent endothelial cells were also characterized by the decreased expression of occludin and claudin-5, which caused the increased permeability of endothelial cell monolayers^30^. The senescence-related reduction in E-cadherin and desmoglein have never been described before.

**Figure 5:**
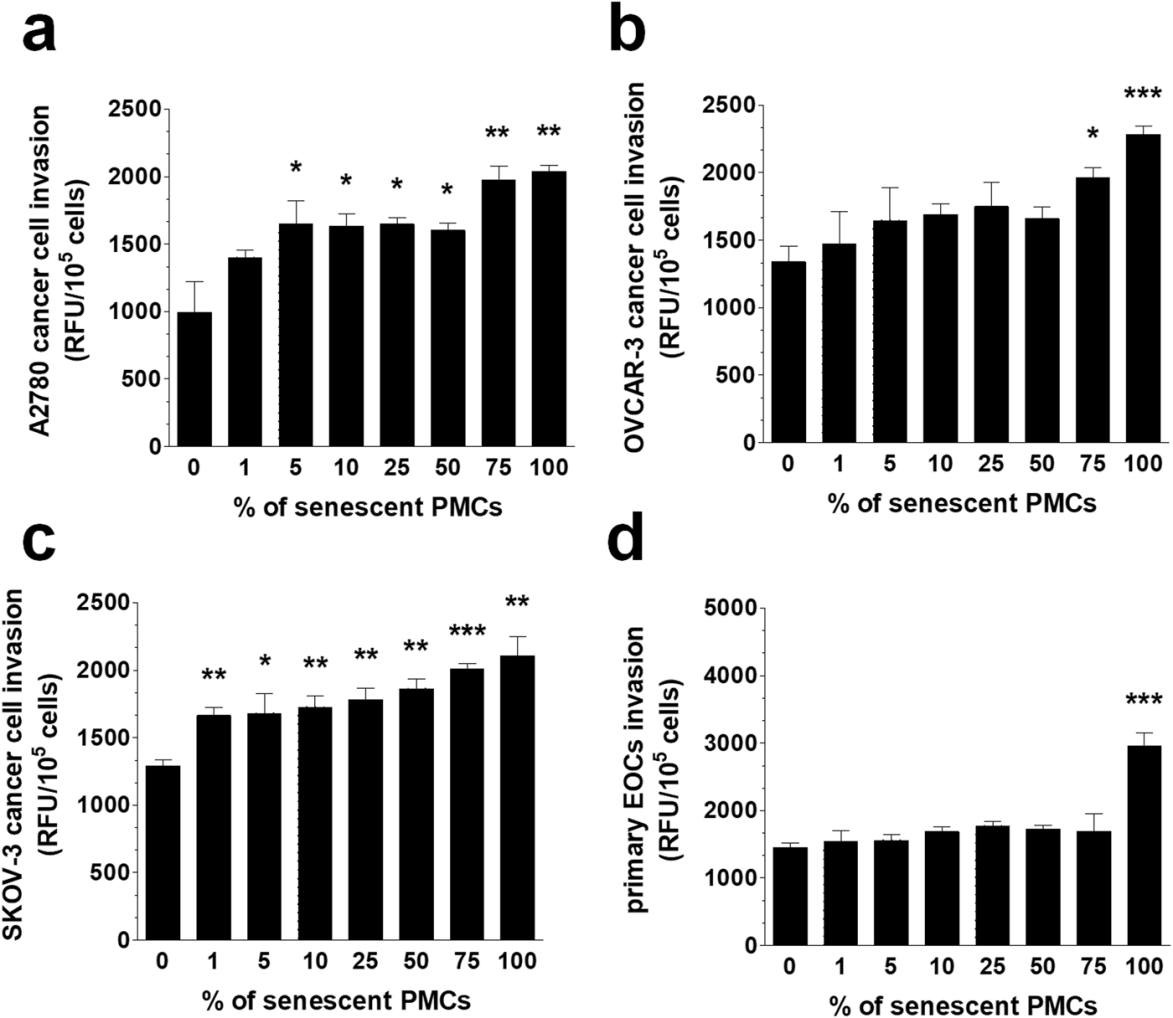
Invasion of the established (**a**–**c**) and primary ovarian cancer cells (**d**) through a layer of PMCs containing an increasing fraction of senescent cells. Experiments were performed with 8 cultures of PMCs obtained from different donors. Cancer cells were used in hexaplicates. The results are expressed as the mean ± SEM. *P < 0.05; **P < 0.01; ***P < 0.001 vs. 0%. EOCs – Epithelial Ovarian Cancer Cells; RFU – Relative Fluorescence Units.

Taking into account above-mentioned observations on endothelial cells, we decided to find out experimentally whether disrupted cell-cell communication due to a reduction of the intercellular junctions in senescent PMCs may influence the invasiveness of cancer cells. To this end we employed an invasion assay in which monolayered PMCs containing different proportions of senescent cells were seeded on the extracellular matrix-mimicking BME substrate. Then, three stable (A2780, OVCAR-3, SKOV-3) and primary EOC cultures were allowed to move towards a chemotactic stimulus generated by a low concentration of serum. The results of this experiment, presented in Fig. 5, revealed that as the proportion of senescent cells increased, more transmesothelial invasion of cancer cells was observed. Interestingly, the stimulatory effect of the senescent cells on the invasion was cell line-specific. In the A2780 cells, invasion was stimulated by the inclusion of a total of 5% senescent cells within the admixture; in the OVCAR-3 cells, the stimulation was seen with a 75% senescent cell admixture; in SKOV-3 cells it was seen with a 1% senescent admixture; whereas in the case of the primary EOCs, the PMCs supported the invasion when the whole population of the PMCs was senescent. We can speculate that these differences may be associated to some extent with the slightly different morphologies of the cancer cells (e.g., the majority of the primary EOCs displayed a spindle-shaped appearance), and they may reflect to some extent the genetic variations among the cell lines, which may also affect the rate of their motility^31^.

Having established that the senescence of PMCs is associated with increased ovarian cancer cell invasion and the decreased expression of the intercellular junctions, we went on to analyze the signaling that links these two phenomena. To this end, we performed experiments in which the expression of the junctional proteins was quantified in pre-senescent PMCs upon their treatment with chemical inhibitors of the pathways recognized to be involved either in the development of the senescent phenotype or in the regulation of the functionality of junctional proteins. These pathways included p38 MAPK^32^, NF-κB^33^, AKT^34^, JNK^35^, SP-1^36^, AP-1^36^, TBX2^37^, and Nkx2-5^37^. Moreover, we aimed to verify whether the deterioration of the junctions could be evoked in an autocrine manner by proteins secreted by the senescent PMCs. In this case, the conditioned medium generated by senescent PMCs was treated with antibodies neutralizing three arbitrarily selected proteins, GRO-1, HGF, and TGF-β1, whose activity has previously been linked with the induction of the senescent phenotype in various cell types^12,38,39^. The idea that the secretome of senescent PMCs may be causatively linked to the down-regulation of the expression of junctional proteins stems from a previous report that demonstrated the capacity of the conditioned medium to support invasion^40^.

**Figure 6:**
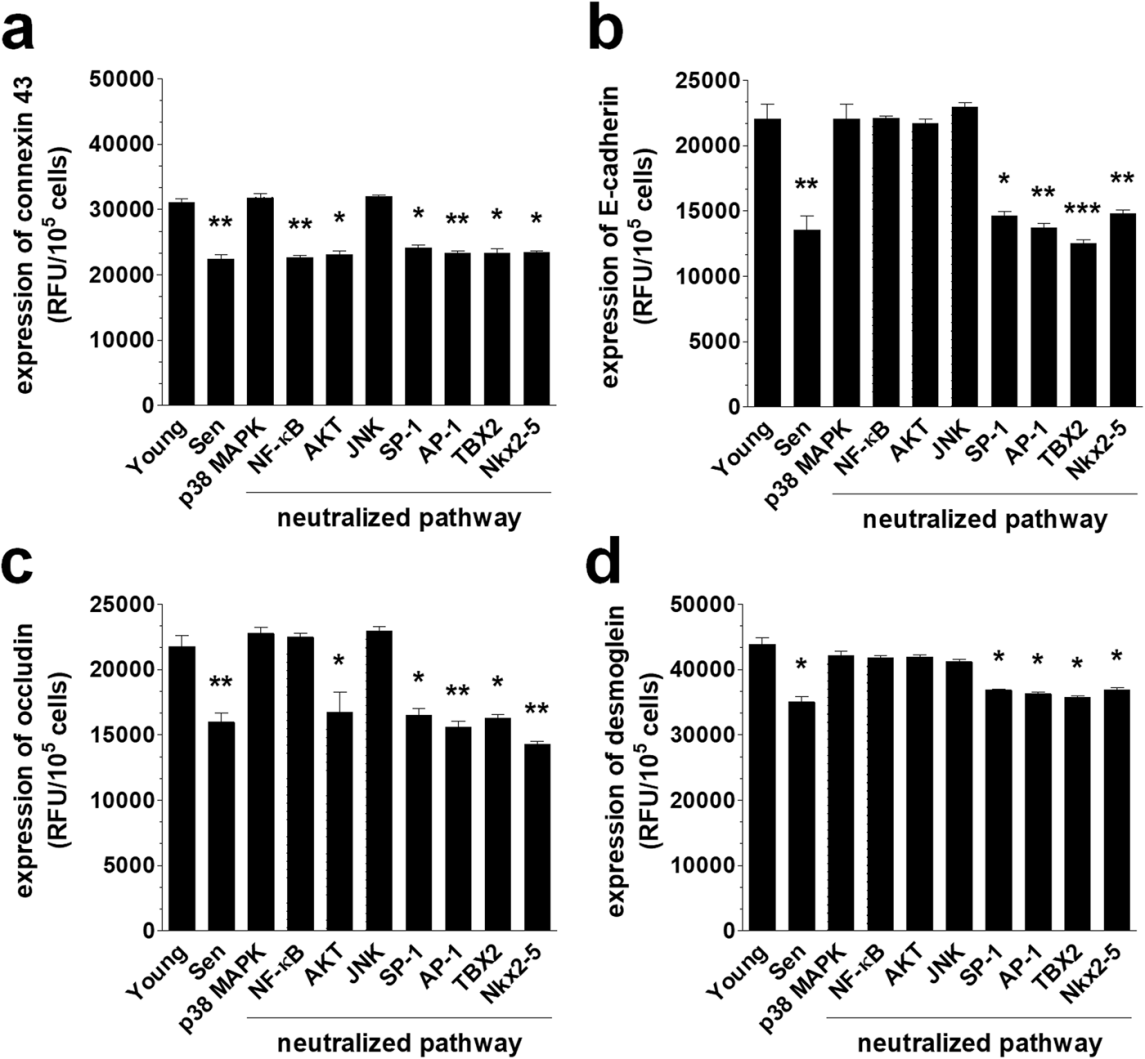
A representation of various pathways involved in the deterioration of junctional proteins in senescent PMCs. Experiments were performed with 6 cultures of PMCs obtained from different donors. The results are expressed as the mean ± SEM. *P < 0.05; **P < 0.01; ***P < 0.001 vs. Young; RFU – Relative Fluorescence Units.

**Figure 7:**
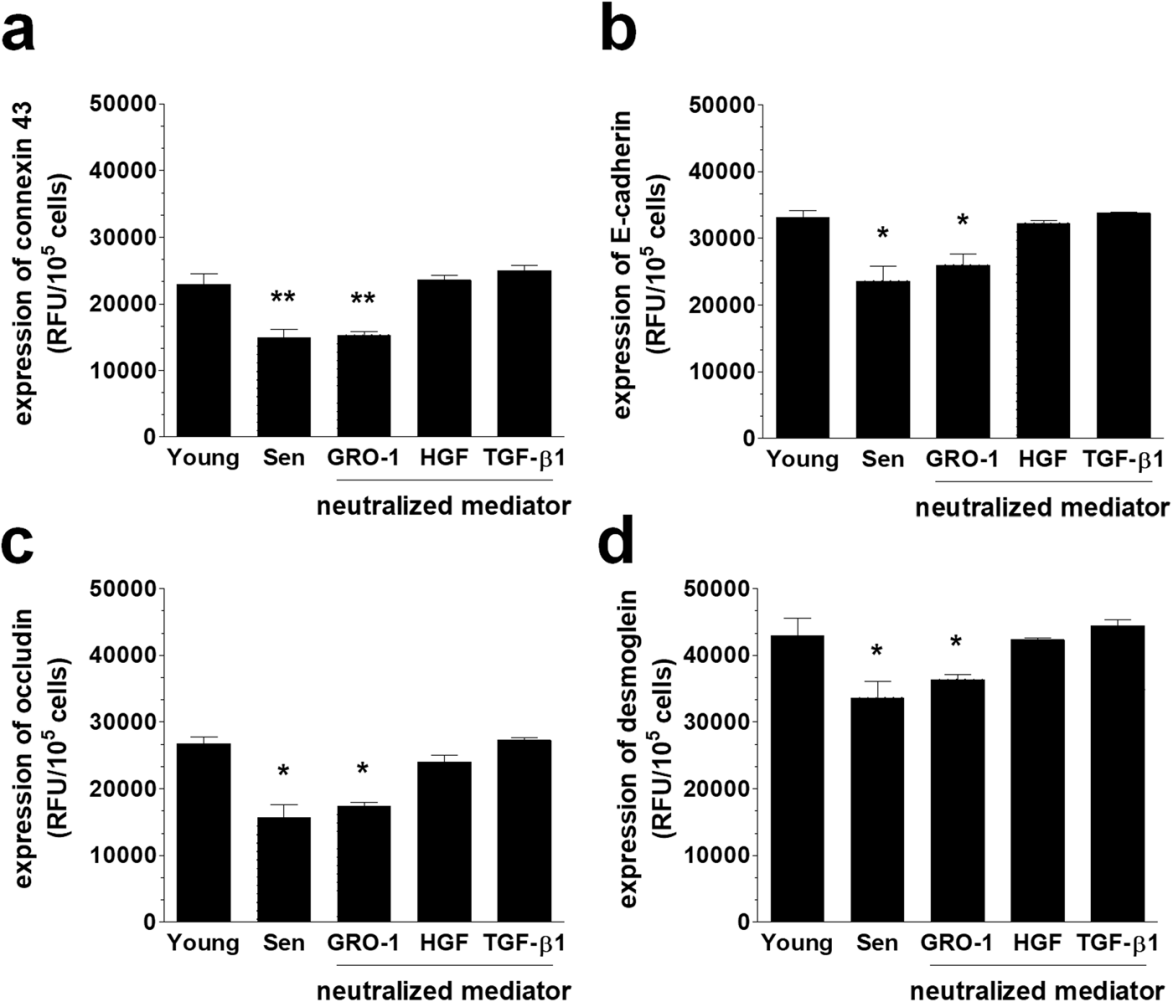
A representation of potential soluble mediators of the deterioration of junctional proteins in senescent PMCs. Experiments were performed with 6 cultures of PMCs obtained from different donors. The results are expressed as the mean ± SEM. *P < 0.05; **P < 0.01 vs. Young; RFU – Relative Fluorescence Units.

**Figure 8:**
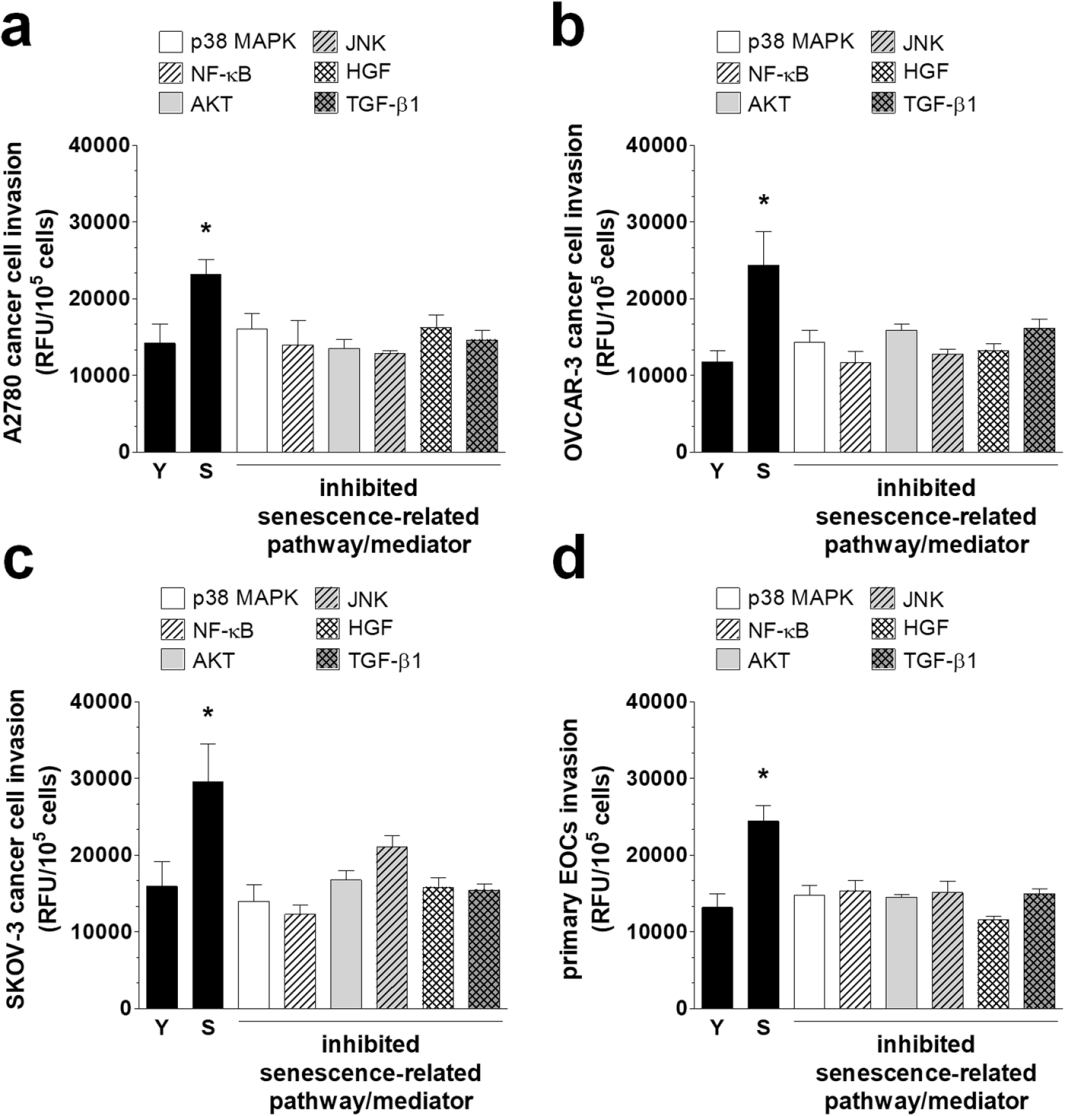
Invasion of the established (**a**–**c**) and primary ovarian cancer cells (**d**) through young (Y) and senescent (S) PMCs, as well as through PMCs in which certain signaling pathways and autocrine mediators, recognized to contribute to a senescence-associated decline in the level of junctional protein, were neutralized. Experiments were performed with 8 cultures of PMCs obtained from different donors. Cancer cells were used in hexaplicates. The results are expressed as the mean ± SEM. *P < 0.05 vs. Young. EOCs – Epithelial Ovarian Cancer Cells; RFU – Relative Fluorescence Units.

Our experiments showed that the senescence-associated decrease in the four tested junctional proteins could be effectively reversed to levels that are characteristic of young cells upon the neutralization of p38 MAPK, NF-κB, AKT, and JNK (Fig. 6). As for the soluble proteins that were released by the senescent cells that could down-regulate the intercellular junctions, HGF and TGF-β1 appeared to play a role (Fig. 7). The significance of these molecules was confirmed by functional tests in which the blockade of the identified signaling and mediatory agents caused a noticeable reduction of the effectiveness of the transmesothelial invasion of ovarian cancer cells (Fig. 8). These findings are in line with observations by other authors who found that p38 MAPK and TGF-β1 contribute to the PMC-dependent exacerbation of the invasive capabilities of ovarian cancer^41^. The findings also agree with another study that showed that TGF-β1 has the capacity to increase ovarian cancer cell invasion via the reduction of E-cadherin levels, due to the elevated activity of transcriptional repressors of this junctional protein, such as Snail, Slug, Twist and ZEB1^42^. NF-κB has also been recognized as being involved in the ovarian cancer metastasis-promoting activity of PMCs^43^. Finally, two papers revealed the role of PAI-1, whose proteolytic activity may be vital for the digestion of the extracellular matrix during cancer cell invasion. This is intriguing because the increased production of PAI-1 by senescent PMCs has also been observed^44^. Another report that is consistent with our results demonstrated the stimulation of ovarian cancer invasion by HGF, which exerted its activity through the down-regulation of thrombospondin-1^45^. Additionally, the roles of AKT and JNK in the regulation of the invasive properties of ovarian cancer have already been demonstrated^46,47^.

## Conclusions

Collectively, our study demonstrated that the presence of senescent PMCs in the peritoneal cavity may contribute to the increased invasiveness of ovarian cancer cells towards the tissue stroma. We showed phenomenologically and mechanistically that this increased invasion might be caused by a senescence-associated decline in several intercellular junction proteins within PMCs. At the same time, we would like to emphasize that our observations, especially those originating from the analysis of ovarian tumor *in vivo*, should be treated with caution as our imagination of cellular events that occurred during various stages of the intraperitoneal tumor formation. For this reason, the usefulness of the *in vivo* results is to some extent limited and their interpretation difficult. Some doubts may be associated, e.g., with the fact that we assumed that ovarian cancer cells did truly invade the mesothelium during very initial phases of cancer cell implantation. However, we cannot rule out the possibility that these mesothelial cells approached there after the tumor formation. To finally solve this important issue, further *in vivo* experiments with laboratory animals should be performed in which much more precise, cell-tracing methods will be employed.

## Material and Methods

### Chemicals

Unless otherwise stated, all chemicals and plastics were from Merck (Darmstadt, Germany). Signaling pathway inhibitors: MG132 (NF-κB), API-1 (AKT), SP600125 (JNK), and 3-Aminobenzamide (AP-1) were from Tocris Bioscience (Ellisville, MO); SB202190 (p38 MAPK) was from Cell Signaling Technology (Danvers, MA); mithramycin A (SP-1) was from Cayman Chemical (Ann Arbor, MI); and peptides blocking TBX2 and Nkx2-5 were from MyBioSource (San Diego, CA). Antibodies neutralizing GRO-1, HGF, and TGF-β1, as well as the control IgY (AB-101-C) were from R&D Systems (Abingdon, UK).

### Tumors from patients with peritoneal ovarian cancer

Tumors excised from the peritoneal cavity of 32 patients with epithelial ovarian cancer (stage III and IV) as well as fragments of tumor-free peritoneum from the same patients were included in the study. The age of patients ranged from 42 to 60 years. The tissues were fixed in 4% formalin and were then processed as described in^11^. The cancer tissue was identified using standard H + E staining. Peritoneal mesothelium located near the cancer cells was identified based on positive stainings of the D2-40, Wt-1, HBME-1, and calretinin with specific antibodies (Dako, Santa Clara, CA). Antigen visualization was performed using an Envision Flex (Dako).

### Cell culture

Primary peritoneal mesothelial cells (PMCs) were isolated by enzymatic disaggregation of the omentum obtained from 8 patients undergoing abdominal surgery (24–30 years old). The cells were propagated as described in^48^ and were identified according to positive staining for the HBME-1 antigen. Senescence of the PMCs was induced by serial passaging until exhaustion of their replicative capacity. The cells were considered senescent when they failed to divide within 4 weeks, and >90% expressed senescence-associated β-galactosidase (SA-β-Gal). In some tests, conditioned medium (CM) from the PMCs was collected^12^.

The ovarian cancer cell lines A2780 and SKOV-3 were purchased from the ECCC (Porton Down, UK), and the OVCAR-3 line was obtained from ATCC (Rockville, MD). The cancer cells were maintained as described in^25^. Primary EOCs were isolated from the tumors removed during cytoreductive surgery from the 8 patients with serous ovarian cancer (stage IV). The detailed procedure of the isolation, identification, and culture of these cells was described in^22^.

### Ethical issues

The procedures involving human subjects (the isolation of tumors, PMCs, and primary EOCs) were in accordance with the Helsinki Declaration of 1975. The study was approved by the Bioethics Committee at the Poznan University of Medical Sciences (consent number 299/17), and all the patients gave their informed consent. The methods were carried out in accordance with the relevant guidelines and regulations.

### Invasion assay

Cancer cell invasion was measured with a Cultrex 96 Well BME Cell Invasion Assay (Trevigen Inc., Gaithersburg, MD). Briefly, PMCs (1 × 10^5^ cells/well) were seeded on the basement membrane extract (BME) to form a monolayer. Then, ovarian cancer cells were added into the upper part of the system (1 × 10^4^ cells/well) on top of the PMCs. The invasion of the cancer cells towards a chemotactic gradient generated by 1% FBS was monitored for 24 h. The fluorescence emitted by the cancer cells was recorded using a Synergy^TM^ 2 plate reader (BioTek Instruments, Winooski, VT) at the 435 nm excitation and 535 nm emission wavelengths.

In some experiments, cancer cell invasion was examined in the presence of an increasing fraction of senescent PMCs. To this end, special co-cultures were prepared in which young PMCs were mixed with senescent cells from the same donor to get desirable percentages of senescent cells in the monolayer.

### Analysis of junctional proteins in cell culture and ovarian tumors

The expression of junctional proteins in cell culture was tested with immunofluorescence using antibodies against connexin 43 (Abcam, Cambridge, UK, 1:100), E-cadherin (Abcam, 1:100), occludin (Novus Biologicals, Littleton, CO, 1:100), and desmoglein (Abcam, 1:10), as described in^22^. As for paraffin sections, immunohistochemistry was performed using antibodies against connexin 43 (Proteintech, Wuhan, China, 1:200), E-cadherin (Dako, 1:200), occludin (Origene, Rockville, MD, 1:150), and desmoglein (Proteintech, 1:500). The staining was visualized using a Novolink Polymer Detection System (Novocastra Reagents, Wetzlar, Germany). Representative pictures were taken using an Axio Vert. A1 microscope (Carl-Zeiss, Jena, Germany). The intensity of the staining was quantified using ImageJ v1.52a (http://rsb.info.nih.gov/ij/). In brief, 20 microphotographs of randomly selected areas of a tumor were taken and analyzed. During the analysis, the area of PMCs was precisely outlined and cells displaying positive color reaction were marked. Then, the area corresponding to positive cells was quantified and expressed as % of the total area of PMCs.

### Detection of senescent cells *in vivo* and *in vitro*

The presence of senescent PMCs was visualized using the cytochemical staining of senescence-associated β-galactosidase (SA-β-Gal), precisely according to the method described by Dimri and colleagues^49^.

### Intervention studies

In some experiments, the expression of junctional proteins in PMCs and the invasion of cancer cells were analyzed in the presence of chemical inhibitors of p38 MAPK (10 µM), NF-κB (10 µM), AKT (50 µM), JNK (10 µM), SP-1 (10 nM), AP-1 (50 µM), TBX-2 (0.165 μg/ml), and Nkx2-5 (0.165 μg/ml), as well as neutralizing antibodies against GRO-1 (800 ng/ml), HGF (1 µg/ml), and TGF-β1 (400 ng/ml). In the case of signaling pathway blockade, pre-senescent PMCs were pretreated with the chemical inhibitors for 4 h, whereas in the case of the neutralization of soluble proteins, the antibodies were added to CM from senescent PMCs 72 h before the study. The specificity and effectiveness of the inhibition/neutralization were verified in the preliminary studies, during which either the concentrations or times of exposure were optimized.

### Statistics

Statistical analysis was performed using GraphPad Prism™ 5.00 (GraphPad Software, San Diego, CA). The means were compared with the Wilcoxon test. The results were expressed as the mean ± SEM. Differences with a P-value < 0.05 were considered to be statistically significant.

## Data Availability

All data analyzed during this study are included in this published article.
